# Structure of the Cyanuric Acid Hydrolase TrzD Reveals Product Exit Channel

**DOI:** 10.1038/srep45277

**Published:** 2017-03-27

**Authors:** Asim K Bera, Kelly G. Aukema, Mikael Elias, Lawrence P. Wackett

**Affiliations:** 1 BioTechnology Institute, University of Minnesota, St. Paul, MN 55108, USA; 2 Department of Biochemistry, Molecular Biology and Biophysics, University of Minnesota, St. Paul, MN 55108, USA

## Abstract

Cyanuric acid hydrolases are of industrial importance because of their use in aquatic recreational facilities to remove cyanuric acid, a stabilizer for the chlorine. Degradation of excess cyanuric acid is necessary to maintain chlorine disinfection in the waters. Cyanuric acid hydrolase opens the cyanuric acid ring hydrolytically and subsequent decarboxylation produces carbon dioxide and biuret. In the present study, we report the X-ray structure of TrzD, a cyanuric acid hydrolase from *Acidovorax citrulli*. The crystal structure at 2.19 Å resolution shows a large displacement of the catalytic lysine (Lys163) in domain 2 away from the active site core, whereas the two other active site lysines from the two other domains are not able to move. The lysine displacement is proposed here to open up a channel for product release. Consistent with that, the structure also showed two molecules of the co-product, carbon dioxide, one in the active site and another trapped in the proposed exit channel. Previous data indicated that the domain 2 lysine residue plays a role in activating an adjacent serine residue carrying out nucleophilic attack, opening the cyanuric acid ring, and the mobile lysine guides products through the exit channel.

Cyanuric acid hydrolases are rare, ancient enzymes undergoing a modern renaissance due to the annual production of more than one billion pounds of *s*-triazine ring compounds that are microbially biodegraded via cyanuric acid as a metabolic intermediate^1^. *s*-Triazine rings are not known to be biosynthesized, but cyanuric acid forms from abiotic chemistry^2^, and their facile synthesis from cheap precursors has spurred industrial production of more than one hundred *s*-triazine compounds. Cyanuric acid, or 1,3,5-triazinane-2,4,6-trione, is a building block for commercial triazines and it is used directly, principally to stabilize hypochlorite for chlorine-based disinfection processes.

The use of more than 100 million pounds of cyanuric acid as a chlorine stabilizer in outdoor pools, spas and fountains has provided new impetus to study the cyanuric acid hydrolases that can enzymatically remove the compound from those waters^3^,^4^. In waters requiring disinfection, chlorine dissipates over time but cyanuric acid is extremely stable, accumulates, and with increasing concentration, it sequesters all of the free chlorine and diminishes its effectiveness in destroying viruses, bacteria and protozoa^5^. In this context, a facile, enzymatic method for removing cyanuric acid from water has been sought.

Cyanuric acid hydrolases are members of a protein family containing only themselves and the barbiturase enzymes, they are found only in microorganisms, and are present in approximately 0.3% of sequenced prokaryotic genomes^6^. The enzyme is proposed to catalyze an opening of the *s*-triazine ring via a nucleophilic serine residue that is activated by a nearby lysine, a mechanistic motif known as a serine-lysine dyad^7^,^8^. In fact, the enzyme contains three serine-lysine dyads and there is some controversy as to the specific serine residue that is most likely to act as a nucleophile^9^,^10^. The active site is highly symmetrical with a single serine-lysine dyad from each of the three domains coordinating the three-fold symmetric substrate. One group has proposed a serine residue in domain one as the most likely nucleophile^10^, and the other group has suggested the serine in domain two to be the putative nucleophile^9^. This different interpretation remains unresolved to date because the three active site serine-lysine dyads have such a high degree of symmetry that discerning differential functions has yet not been possible. Regardless of which serine is ultimately shown to be the nucleophile, the resultant serine ester thus formed would be expected to undergo hydrolysis to generate carboxybiuret (Fig. 1). Previous studies have shown that carboxybiuret undergoes spontaneous decarboxylation to biuret with a half-life of several minutes at neutral pH^6^. Given the chemical instability of carboxybiuret, it has not been possible to determine if there is some enzyme assistance in the decarboxylation reaction, and this is one of the issues addressed in the present study.

X-ray structures for cyanuric acid hydrolases are crucial for using the enzymes for treating disinfection waters^1^. The enzyme TrzD, a cyanuric acid hydrolase from *Acidovorax citrulli*^11^ shows 58% and 50% sequence identity to the previously-studied enzymes from *Pseudomonas* sp. ADP^12^ and *Azorhizobium calindulans*^9^, respectively, and TrzD is reported to have a *k*_*cat*_ that is an order of magnitude greater than other known cyanuric acid hydrolases^11^. Given the importance of using a high *k*_*cat*_ enzyme in commercial applications, and for obtaining insights into the outstanding mechanistic questions, the present study focused on determining the X-ray structure of TrzD. In the course of the study, we observed an unusual orientation of the second-domain lysine that interacts with the proposed catalytic second-domain serine. This observation simultaneously provided support that the second domain serine serves as the active site nucleophile and it gave evidence for a product exit channel. Moreover, the presence of trapped carbon dioxide in the enzyme is consistent with the view that the enzyme assists in the decarboxylation of carboxybiuret, and the cell does not rely on a spontaneous decarboxylation to produce the next intermediate, biuret, in the cyanuric acid biodegradation pathway (Fig. 1).

## Methods

### Protein Expression and Purification

The full-length *trzD* gene, cloned in pET28b( + )vector^6^, was expressed in *Escherichia coli* BL21 (DE3) that was grown in LB medium (10 g Bacto tryptone, 5 g yeast extract, 10 g NaCl per liter) supplemented with 50 ug/ml kanamycin. Cells were grown at 37 °C and induced with 0.5 mM of isopropyl β-D-1-thiogalactopyranoside (IPTG) for 30 hours at 20 °C. After collection by centrifugation (6400 × g, 10 min, 4 °C), the cells were resuspended in buffer A (50 mM Tris–HCl pH 7.2, 200 mM NaCl). Cells were lysed by three passes at 1000 p.s.i. through a French press, and the cell lysate was centrifuged at 48,000 × g for 45 min at 4 °C. After centrifugation, the cell lysate was applied onto a column packed with a Ni–NTA resin (Thermo Scientific) that had been equilibrated with the binding buffer. After washing, the bound protein was eluted from the column with a gradient using elution buffer B (50 mM Tris–HCl pH 7.2, 200 mM NaCl, 200 mM imidazole). For further purification, gel filtration chromatography was performed using a HiLoad™ Superdex-200 16/600 gel-filtration column (GE Healthcare) equilibrated with Buffer A. The enzyme was concentrated to 12 mg/ml and stored at 4 °C until used.

### Crystallization

The protein was crystallized with its natural substrate cyanuric acid by hanging drop, vapor-diffusion experiment in which equal volume of protein (12 mg/ml concentration) and reservoir solution were mixed and allowed to equilibrate against the reservoir at 20 °C. The final concentration of cyanuric acid was 10 mM in the crystallization drop. The initial crystallization hit contained 0.05 M ammonium sulfate, 0.05 M BIS-TRIS pH 6.5, and 30% pentaerythritol ethoxylate (15/4 EO/OH). Needle-shaped crystals appeared in 48 hours. Two week old crystals were harvested and flash-cooled in liquid nitrogen without additional cryoprotectant, since 30% pentaerythritol ethoxylate (15/4 EO/OH) serves as good cryoprotectant. A single crystal was used for data collection.

### Data Collection and Processing

The flash-cooled crystal was diffracted at beamline 23-ID-B in the Advanced Photon Source (APS) at 1.03322 Å wavelength with 0.5 degree oscillation per frame. A MAR CCD300 detector was used to collect 270 frames at a distance of 250 mm from the crystal. All data were processed and reduced using the HKL2000 package^13^. Crystals grown with cyanuric acid belonged to I222 space group with unit cell dimensions of 67.7, 90.8, and 120.3 Å and diffracted to 2.19 Å. Crystal showed an average mosaicity of 0.33 degree. The asymmetric unit contained one monomer with a Matthews coefficient of 2.32 with 47% solvent content.

### Structure Determination and Refinement

Molecular replacement was performed using the software MOLREP^14^ with the monomer of AtzD (PDB Code 4BVQ)^10^ as the search model. Preliminary phases derived from the initial atomic models showed strong electron densities for ligands in the active site for crystals grown with cyanuric acid. Iterative cycles of model building and refinement were conducted using COOT^15^ and PHENIX^16^ alternatively against a dataset at 2.19 Å resolution. Refinement of the model against X-ray data was carried out until high quality electron density maps and satisfactory model statistics were achieved. MolProbity^17^,^18^ and PROCHECK^19^ were used for structure quality analysis. For the final structures, the Ramachandran plot showed that 98% of the residues are in the most favored region and the other 2% are in the allowed region. A summary of the data collection and model refinement statistics is shown in Table 1.

## Results

### Overall Structural Features

**Figure 1:**
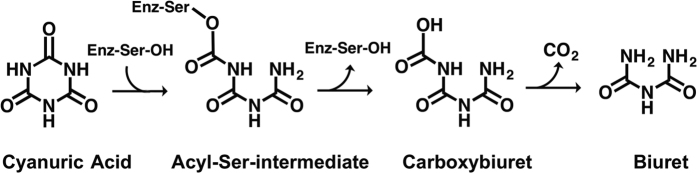
Proposed reaction scheme of cyanuric acid hydrolase.

Cyanuric acid hydrolases are a unique class of proteins structurally, showing a pseudo three-fold symmetry. They show significant divergence, most share ~40–60% sequence identity in pair-wise alignment with other members of the cyanuric acid hydrolase/barbiturase protein family. Cyanuric acid hydrolase proteins differ from each other largely in their respective kinetic parameters and thermal stability^6^,^20^. The data were phased using coordinates from the X-ray structure of AtzD (cyanuric acid hydrolase from *Pseudomonas* sp. ADP) (4BVQ). The crystallographic asymmetric unit contained one TrzD molecule. The product-bound structure was refined to a resolution of 2.19 Å, with R_work_ and R_free_ values of 0.15 and 0.20, respectively, yielding high quality electron density maps. We could model the N-terminal His-tag. The crystallographic data collection and refinement statistics are listed in Table 1, and the coordinates and structure factors have been deposited as Protein Data Bank entry 5T13. Structural comparison of TrzD with available cyanuric acid hydrolase structures shows 58% sequence identity and 1.0 Å rmsd (on 364 carbon alpha) with AtzD and 50% sequence identity and 1.4 Å rmsd (on 351 carbon alpha) with ACAH (a cyanuric acid hydrolase from *Azorhizobium calindulans*). The next nearest structural homolog is chorismate mutase (4BPS) with 11% sequence identity and 4.0 Å rmsd (using the DALI server)^21^. An examination of the active site revealed, unexpectedly, the active site lysine residue in domain 2 (Lys163), that in other structures paired with the proposed nucleophilic serine (Fig. 1), exhibited a significant displacement out of the active site, and opening a clear channel from the buried active site to the protein surface. Moreover, the difference Fourier map (F_o_ − F_c_) clearly showed electron density corresponding to two CO_2_ molecules and Lys163 within the TrzD structure (Figs S1 and S2). As expected, since CO_2_ is a co-product, one is located in the active site, while the second one was observed at the interface of domain 2 and 3, proposed here as the exit channel for product release.

**Figure 2:**
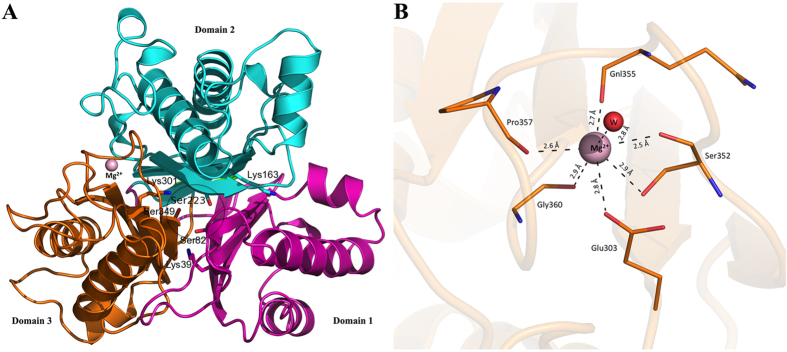
(**A**) The three domains of TrzD shown in cartoon diagram: Domain 1 in magenta, Domain 2 in cyan and Domain 3 in orange, respectively. The active site is highlighted by a black circle. Active site serines and lysines are shown in stick. Mg^2+^ ion is shown as the pink sphere. (**B**) Coordination of Mg^2+^ ion in a conserved metal binding flap in Domain 3.

TrzD is a 370 amino acid, ~39.5 kDa protein that exhibits a complex α/β structure characteristic of the cyanuric acid hydrolase family enzyme. The active site is situated inside a core composed of 4 β-strands from each of the three-isostructural domains. Surface exposed elements are helices and loops. TrzD has a short β-hairpin and 9 significant helix-helix interactions. Domain 1 is composed of 107 (1 to 107), domain 2 is composed of 137 (113 to 249) and domain 3 is composed of 115 (255 to 369) residues. Each domain starts and ends with two antiparallel β-strands. Domain 2 has a 15 residue long extra helix, which is not present in the other two domains, making it slightly bigger. Residues important for catalysis, i.e., Ser, Lys and Arg residues from each domain, showed similar spatial disposition, when superimposed. The three structurally homologous domains form a pseudo-three-fold internal symmetry in the TrzD monomer (Fig. 2A). The latter is likely selected by nature to accommodate the three-fold symmetric substrate cyanuric acid. Interestingly, there are three psi loops, one in each domain of TrzD and all active sites serines are at the beginning of the corresponding psi loop. Psi loops are rare elements in protein structures. The active site cleft is highly organized; three serines and three lysines originate from two parallel beta-strands while their cognate arginines originate from the longest helix from the back. The other two beta-strands at the active site core are longer and running antiparallel. A small 2:2 beta-hairpin is observed in domain 3, near the metal-binding flap.

### Crystal Contacts

Based on size exclusion chromatography and dynamic light scattering, TrzD forms a stable tetramer in solution. An analysis of present structure by the PISA server (PISA v1.51 [22/09/2014]) predicted an average solvation free energy of −70 kcal/mol for tetramer formation. The average solvent-accessible surface area of monomeric units buried upon assembly formation and solvent-accessible surface area for a tetramer is 12250 and 51030 Å^2^ respectively, supporting the conclusion that TrzD exists largely as a tetramer in solution. Domains 1 and 2 do not take part in tetramer assembly, nor are they in crystal contact. Domain 1 and domain 3 form six hydrogen bonds between them. Arg314 of domain 3 forms three hydrogen bonds, one with the Met10 main chain carbonyl oxygen and the other two with side chain oxygens of Glu41. Ser321 forms two hydrogen bonds with Val46 and Asn47 of domain 1. The Asp322 side chain oxygen is hydrogen bonded with Asn43. Domain 2 and 3 form three hydrogen bonds. Arg147 and Arg250 from domain 2 form hydrogen bonds with Glu309 and Asp290 side chain oxygen atoms. Arg147 forms another hydrogen bond with the Ala370 main chain carbonyl oxygen. Crystal contacts were analyzed using the LIGPLOT program^22^. Superimposition of ACAH, AtzD structure on TrzD structure and the tetramer assembly are shown in Figs S3 and S4.

### Conserved Metal Binding Site

A conserved metal-binding loop sequence **GGxEHQGPxGG** is identified throughout the cyanuric acid hydrolase/barbiturase protein family. Ser/Ala/Gly residues can substitute in the first X and the second X can be a Ala/Asp/Pro/Ser residue (Fig. S5). This conserved metal binding loop is situated ~13 Å away from the active site and has not been implicated in the catalytic mechanism. The present structure has an Mg^+2^ bound and makes 6-contacts with neighboring residues and one with a solvent molecule (2.8 Å). Mg^+2^ interacts with the backbone carbonyl oxygen of Ser352 (2.5 Å), Gln355 (2.7 Å), Pro357 (2.6 Å) and Gly360 (2.9 Å) and the side chain oxygens of Glu303 (2.8 Å) and Ser352 (2.9 Å). Glu303 is the only residue to interact with the metal ion that is not in the conserved loop (Fig. 2B). The metal binding site formed a uniform alcove in domain 3 and does not interact directly with domain 2.

### Dynamic Nature of Second Domain Lysine163

**Figure 3:**
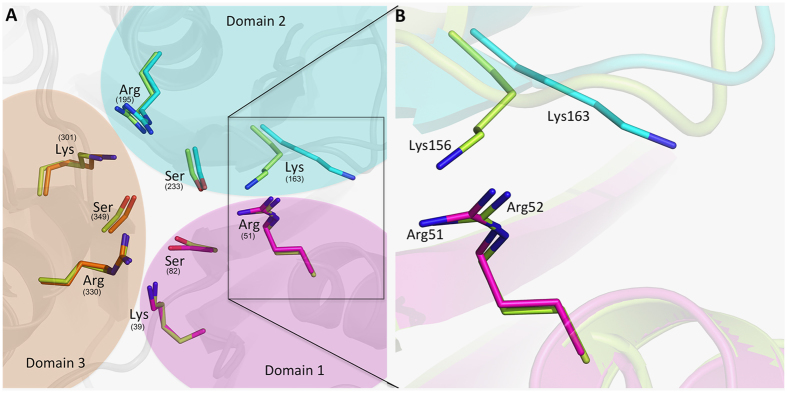
(**A**) Overlay of TrzD on ACAH, ACAH cartoon shown in lime, TrzD carton shown by three domain in three different colors; Domain 1 – magenta, domain 2 – cyan and domain 3 – orange. Three active site lysines, arginines and serines are shown in stick representation with respective domain color. (**B**) TrzD Lys163 and ACAH Lys156 zoomed.

**Figure 4:**
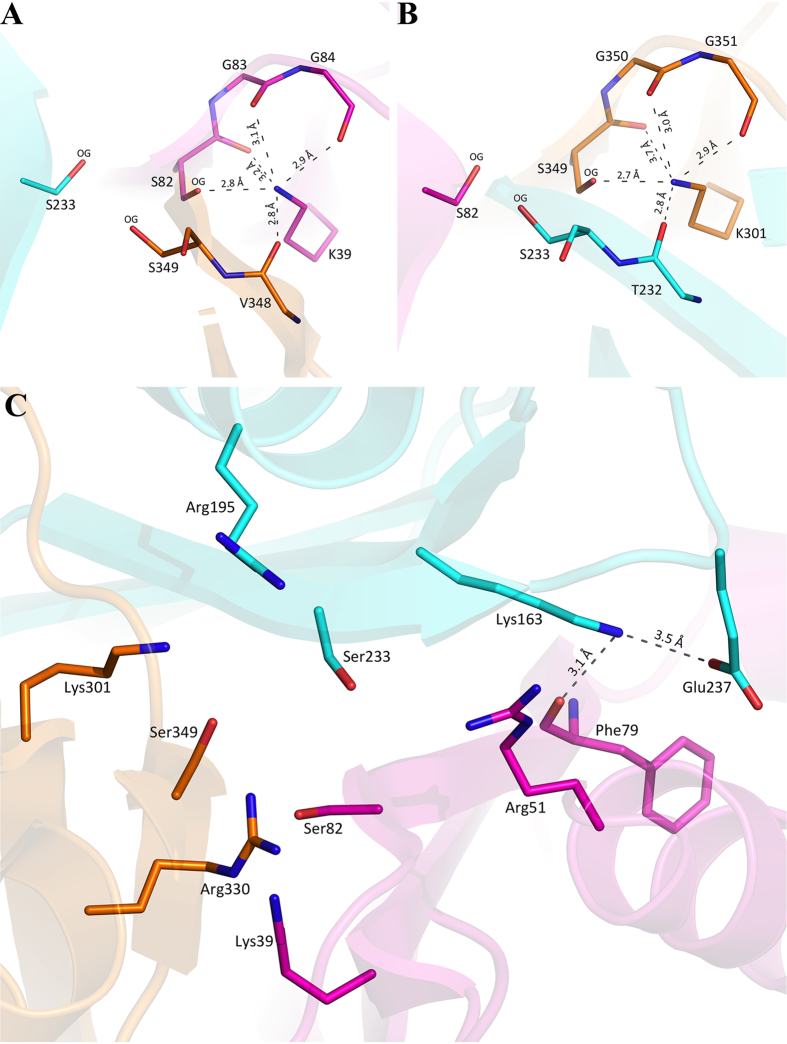
Domain 1 and domain 3 active site lysine’s conserved interaction and anchored tightly in the active site. (**A**) Domain 1, Lys39, (**B**) Domain 3, Lys301. (**C**) Lys163 in Domain 2 stabilized by two hydrogen bonds shown by dashes.

An unusual displacement of the 2^nd^ domain catalytic lysine residue, away from the active site core and with strong electron density, was observed (Fig. 3). The movement does not affect the unique three -domain organization that coordinates and activates the three-fold symmetric substrate cyanuric acid in cyanuric acid hydrolases. The movement of Glu235, Leu238 and Glu242 near the domain 2-Lys163 provides more space for opening to the protein surface, but without extensive main-chain movement. By contrast, the active site lysine residues in domain 1 and 3 are strongly anchored and remain localized in the catalytic center (Fig. 4A,B). The average B-factor of the 2^nd^ domain’s Lys, Ser and Arg are higher than those of domain 1 and 3. In domain 1, Lys39 is hydrogen bonded to Gly83, Gly84, & Val348 main chain carbonyl oxygen atoms, and with the Ser82 main-chain carbonyl oxygen and side-chain oxygen. Similarly, Lys301 of domain 3 is hydrogen bonded with the Ser349 main chain carbonyl oxygen and side chain oxygen and two consecutive glycine main chain oxygen atoms and the Thr232 side chain carbonyl oxygen. This is a unique conserved signature interaction of active site lysines observed in all other available CAH structures^9^,^10^. In contrast, the mobile Lys163 has less extensive neighboring interactions, limited to the main chain carbonyl of Phe79 (3.1 Å) and the side chain oxygen of Glu237 (3.5 Å) as shown in Fig. 4C.

### Active Site CO_2_ Binding Site

**Figure 5:**
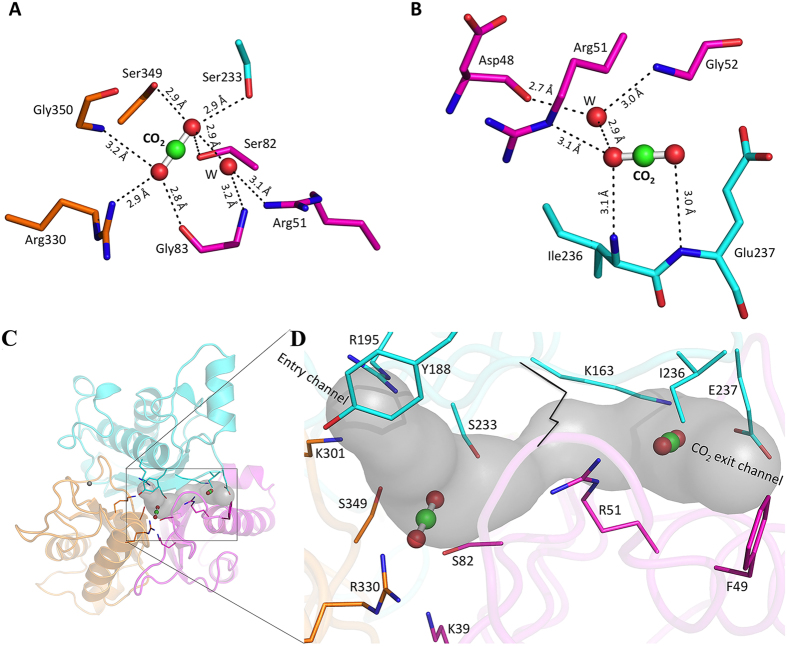
(**A**) Carbon dioxide at the active site. Active site residues are also shown in stick and carbon dioxide molecules in ball-and-stick representation. (**B**) Carbon dioxide at the exit channel. One water molecule in each position is hydrogen bonded with carbon dioxide. (**C,D**) Entry and exit channel: Residues are also shown in stick and carbon dioxide molecules in ball-and-stick representation. Black line shown in panel D, is the Lys156 position, which blocked the exit channel in ACAH structure.

Density analysis clearly revealed two molecules of the co-product, carbon dioxide, with one in the active site and the other in the exit channel. The active site CO_2_ was observed to be within a core made by tight clustering of three serines, three arginines and two lysines. The CO_2_ molecule is stabilized by H-bonds with the side chain oxygen of Ser82, Ser233 and Ser349 (~2.9 Å), and the backbone amide nitrogen of Gly350 (3.3 Å), backbone carbonyl oxygen of Gly83 and also with a side chain nitrogen of Arg330 (Fig. 5A). A solvent molecule is also visible in the vicinity of carbon dioxide at a distance of 2.9 Å.

### Domain Interface CO_2_ Binding Site

Unexpectedly, an unambiguous density corresponding to a second bound CO_2_ was observed in the domain interface, sandwiched between domain 1 and domain 2 near the surface. The oxygen atoms of the CO_2_ molecule were stabilized by two paired H-bonds with the side chains of Arg51 and Glu237 from domain 1 and domain 2, respectively, with additional stabilization by the hydrophobic residue Ile236 backbone amide N and Glu237 backbone amide N (Fig. 5B). The CO_2_ proximal oxygen is also H-bonded to a solvent molecule at 2.9 Å distance. On the basis of only structural observations, the domain interface-bound CO_2_ would appear to be less stable than the active site CO_2_, suggesting it may be in a position penultimate to release from the proposed exit channel.

### Entry and Exit Channels

The surface of the monomer has two cavities that could provide access to the active site for substrate entry and/or product release. One is shorter, around 10 Å from the surface, and is formed by Cys45, Tyr188, Met191, Arg195, and Thr326. Cys45, Tyr188, and Thr326 are proposed to be responsible for controlling substrate entry (Fig. 5C,D). Another clear channel was observed between the domain 1 and domain 2 interface that is longer (~15 Å) than the entry channel and formed by the displacement of active site Lys163. The terminal N-atom of this second domain Lys is hydrogen bonded to the adjacent Met81 and Ser233, in known cyanuric acid hydrolase structures^9^,^10^, and therefore does not open up into what we proposed here to be the exit channel. Arg51, parallel to Lys163 also helps to maintain the exit channel while Ile236, Glu237 and Gly52 form the outer surface of the exit channel. A CO_2_ molecule is bound to this channel, supporting the exit channel concept.

## Discussion

Previously reported cyanuric acid hydrolase structures have three pairs of Ser-Lys-Arg residues clustered in a closed active site pocket of around 155 Å^3^ 23. Adjacent Ser-Lys residues have been proposed to serve as potential catalytic dyads^10^. The TrzD structure described here is the first study identifying the flexibility of the domain 2 lysine and observing the corresponding residue not in close proximity to its cognate serine. In the TrzD structure uniquely, Lys163, which is conserved in all cyanuric acid hydrolases, was re-oriented toward the surface, opening up an exposed channel between the domain 1 and 2 interface. Here we propose this to be the product exit channel. In all other cyanuric acid hydrolase structures, the terminal nitrogen atom of the second domain active site lysine was hydrogen bonded to the side-chain oxygen of its cognate serine, serving to close off the active site^9^. The displacement of Lys163 toward the surface, observed here, does not affect the conserved three-fold symmetry of the global structure. This movement of Lys163 is made possible by the relatively low contact density of this key active site residue.

We note that this conformational change seems physically possible in other cyanuric acid hydrolases, for instance ACAH, where the corresponding lysine residue has room to move toward the surface, as is observed here in the structure of TrzD. It is unusual for an active site residue that participates in the reaction chemistry to have such freedom of movement^24^,^25^. As shown in Fig. S6, superimposition of TrzD with the ACAH structure shows that the Lys156 of ACAH could move similarly as Lys163 of TrzD. On the other hand, the other two active site lysines are anchored tightly and remain localized in the catalytic center forming four conserved hydrogen bonds each as shown in Fig. 4A,B. The Ser-Gly-Gly consensus sequence in each of the two domains, namely 1 and 3 has formed an identical geometry and interaction with their cognate lysine residues.

The refined structure also revealed, unexpectedly and unambiguously, one CO_2_ molecule in the active site. The ligand is bound at the center of the β-barrel in the catalytic site and has occupancies and B-factors fully compatible with the mobility of the surrounding atoms (for CO_2_ and protein molecule are 42 and 40 Å^2^ respectively, Fig. S4).

The new conformation of the Lys163 opens up a new channel connecting the active site with the protein surface. This proposed exit channel is overall hydrophobic in nature, and the electronic density maps reveal unam biguously a second bound CO_2_ molecule that sits in the tunnel. In the view shown in Fig. S7, CO_2_ is visible in the exit tunnel only 3 Å from the surface. Ile236 and Glu237 side-chain moved significantly in comparison to ACAH structure to form the channel for product exit, though their backbone movement is negligible. Phe49 also has some role to the exit channel opening in TrzD during product release, since the bulky side chain has moved.

A common motif in other protein-CO_2_ binding sites is the presence of charged basic residues and a solvent molecule that is hydrogen bonded with CO_2_ oxygen atoms^26^,^27^. In TrzD, a side chain NH_2_ of Arg330 forms a hydrogen bond with the active site CO_2_ and Arg51 is hydrogen bonded to the exit channel carbon dioxide. The Arg51 side chain NH_2_ forms a water-mediated hydrogen bond with the other oxygen of the active site carbon dioxide (Fig. 5A,B). In the case of the active site CO_2_, ligating residues belong to the core beta-sheets, only Arg330 is an exception and comes from a 3^rd^ domain helix. In case of exit channel CO_2_, ligating residues belong to a loop arising at the end of the 2^nd^ domain similar to what has been observed in other CO_2_-binding proteins^26^,^27^. Curiously enough, only Arg51 derived from the 1^st^ domain helix. Comparison with available CO_2_ bound protein structures revealed that the protein-based CO_2_ bonding is guided by acid/base interaction rather than hydrophobic/philic interactions^26^,^28^. Preferences of secondary structural elements more amenable to CO_2_ binding are loops and beta-sheets as opposed to helices.

The displacement of a catalytic lysine residue of domain 2, away from the active site core, suggests its dynamic nature during the reaction cycle. First, after cyanuric ring opening and hydrolysis of the enzyme-ester intermediate, carboxybiuret forms and decarboxylation is enzyme-assisted, producing biuret and carbon dioxide. The displacement of the domain 2 lysine then opens up a channel that allows product release. *In silico*, carbon dioxide remained bound, thus revealing the exit channel. By this model, domain 2 lysine’s role in catalysis is not limited to activating catalytic serine to open the ring but also guides products towards the exit channel.

## Additional Information

**Accession Codes**: Coordinates and structure factors have been deposited in the Protein Data Bank with the accession code of 5T13.

**How to cite this article**: Bera, A. K. *et al*. Structure of the Cyanuric Acid Hydrolase TrzD Reveals Product Exit Channel. *Sci. Rep.* **7**, 45277; doi: 10.1038/srep45277 (2017).

**Publisher's note:** Springer Nature remains neutral with regard to jurisdictional claims in published maps and institutional affiliations.

## Supplementary Material

Supporting Data

## Figures and Tables

**Table 1:** Data Collection and Refinement Statistics.

	TrzD-product
Space group	I222
Cell parameters (*a, b, c)* (Å)	67.59, 90.69, 120.07
Resolution (highest shell)(Å)	29.45–2.19 (2.26–2.19)
No. of measured intensities	106693
No. of unique reflections	19361
Redundancy	5.5
Completeness (last shell) (%)	99.8 (97.9)
*I*/σ(*I*) (last shell)	17.2 (2.6)
*R*_merge_ (last shell)^a^	0.089 (0.632)
CC_1/2_	0.99
CC*	0.98
**Refinement Statistics**	
Resolution range (Å)	29.45–2.19
No. of reflections used	36528
No. of protein atoms	2833
No. of waters	172
*R*_work_^b^	0.15
*R*_free_^c^	0.20
rmsd bond lengths (Å)	0.010
rmsd bond angles (°)	1.012
Ramachandran Favored/allowed Outlier (%)	98.00/2.00 00.00
Average B_factors_	
protein	39.98
ligand	42.09
metal	22.86
water (Å^2^)	35.50

^a^*R*_merge_ = Σ |I − (I)|/ΣI, where I is the intensity of an observed reflection and (I) is the average intensity of multiple observations.

^b^*R* = Σ||*F*_obs_| − |*F*_cal_||/Σ|*F*_obs_|.

^c^*R*_free_ = Σ||*F*_obs_| − |*F*_cal_||/Σ|*F*_obs_|, where *F*_obs_ is from a test set of reflections that are not used in structural refinement.
