# Supplementary material for: Structure of the Cyanuric Acid Hydrolase TrzD Reveals Product Exit Channel
Source: Sci Rep. 2017 Mar 27;7:45277. doi: 10.1038/srep45277 (PMC5366886; doi:10.1038/srep45277)
Supplement: Supporting Data [file srep45277-s1.doc]

**Structure of the Cyanuric Acid Hydrolase TrzD**

**Reveals Product Exit Channel**

Asim K Bera^a^, Kelly G. Aukema^a^, Mikael Elias^a,b^ and Lawrence P. Wackett^a,b^*

^a^BioTechnology Institute and ^b^Department of Biochemistry, Molecular Biology and Biophysics. University of Minnesota, St. Paul, MN 55108, USA.

**Supporting Figure Legends**

**Figure S1: A)** *Fo-Fc* omit map contoured at 3.0 σ level, depicted in green mesh on carbon dioxide. **B)** *2Fo-Fc* electron density contoured at 1.0 σ level, depicted in blue mesh on carbon dioxide, shown in ball-and-stick.

**Figure S2:** Simulated annealing *Fo-Fc* omit map over Lys163 contoured at 3.0 σ level.

**Figure S3:** Superposition of ACAH, AtzD and TrzD in limon, magenta and cyan respectively. The metal ion shown in grey sphere.

**Figure S4:** Locations of the ordered/disordered residues in TrzD tetramer. The main chains are color-ramped from blue to red for *B*-factors from approximately 25 to 93 Å^2^. The higher *B*-factor main chains are also shown in the thicker lines. Domain 3 is most stable; domain 1 is less stable than domain 2 in terms of main chain B-factors. Carbon dioxides are shown in spheres.

**Figure S5:** Domain 3 sequence alignment near metal binding flap. Conserve sequence of metal binding loop in cyanuric acid hydrolase/barbiturase family shows by an arrow.

**Figure S6:** Superimposition of TrzD in cyan on ACAH in lime. **A)** L156 of ACAH (lime) H-bonded to two main chain carbonyl oxygens of S227 & M78 and with S226 OG. There is space for L156 to move in the orientation of TrzD L163 (shown in cyan), and if L156 moves nearest atom distances are 4 Å and more. **B)** 90° rotation along y-axis.

**Figure S7:** Electrostatic potential over TrzD surface. Carbon dioxide shown in the exit channel.


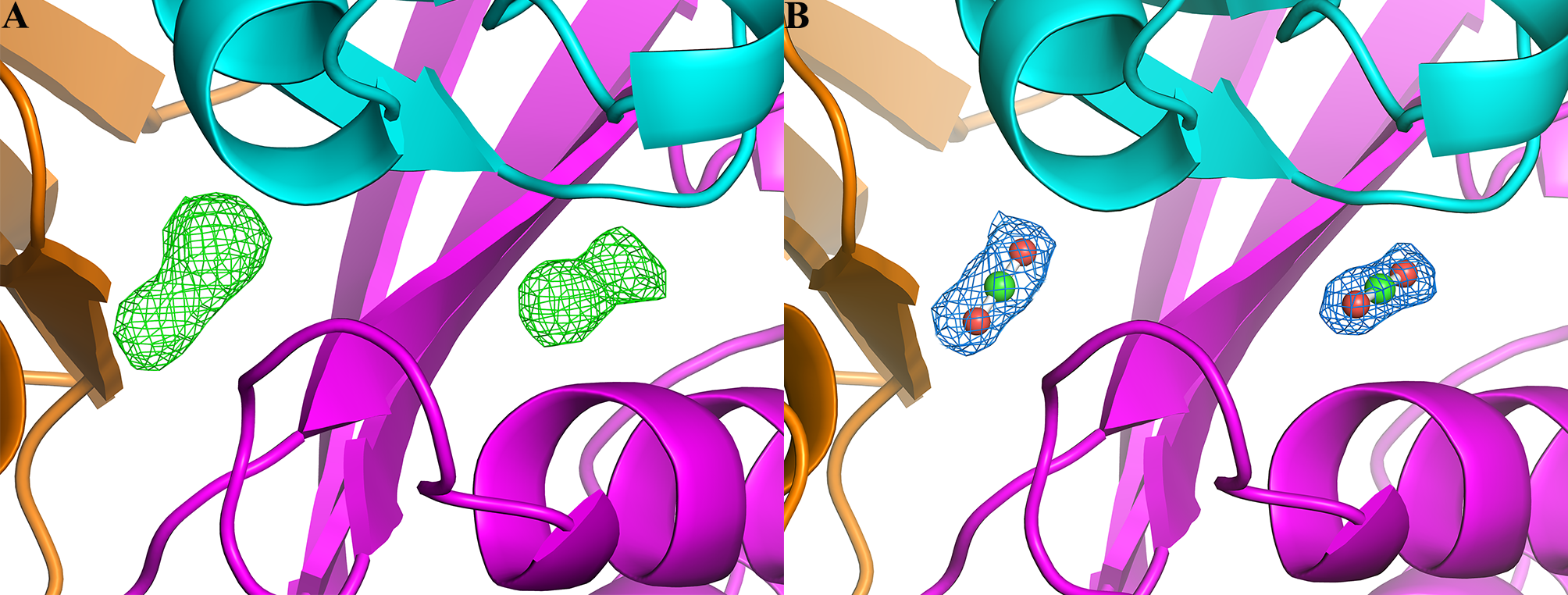


**Figure S1: A)** *Fo-Fc* omit map contoured at 3.0 σ level, depicted in green mesh on carbon dioxide. **B)** *2Fo-Fc* electron density contoured at 1.0 σ level, depicted in blue mesh on carbon dioxide, shown in ball-and-stick.


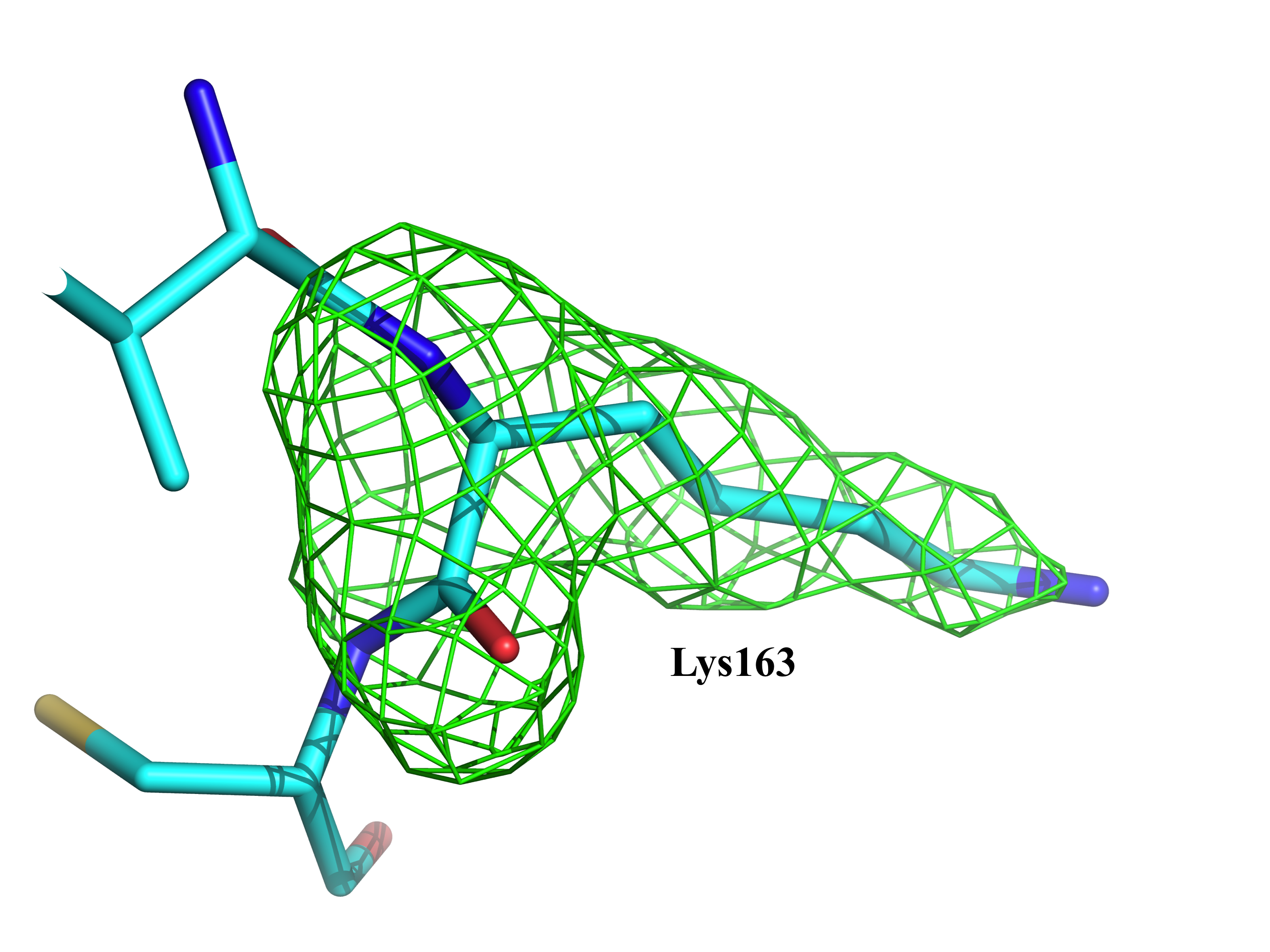


**Figure S2:** Simulated annealing *Fo-Fc* omit map over Lys163 contoured at 3.0 σ level.


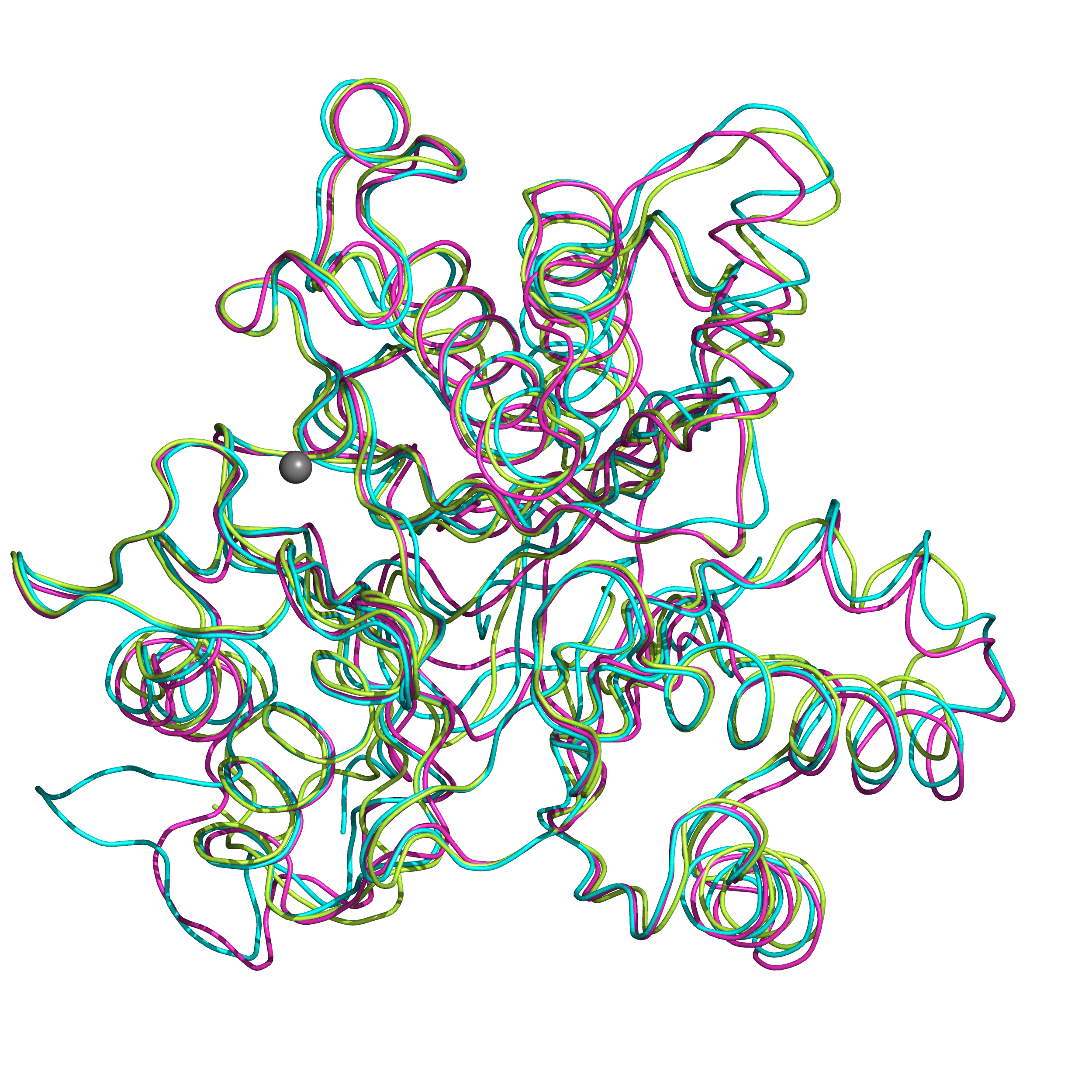


**Figure S3:** Superposition of ACAH, AtzD and TrzD in limon, magenta and cyan respectively. The metal ion shown in grey sphere.


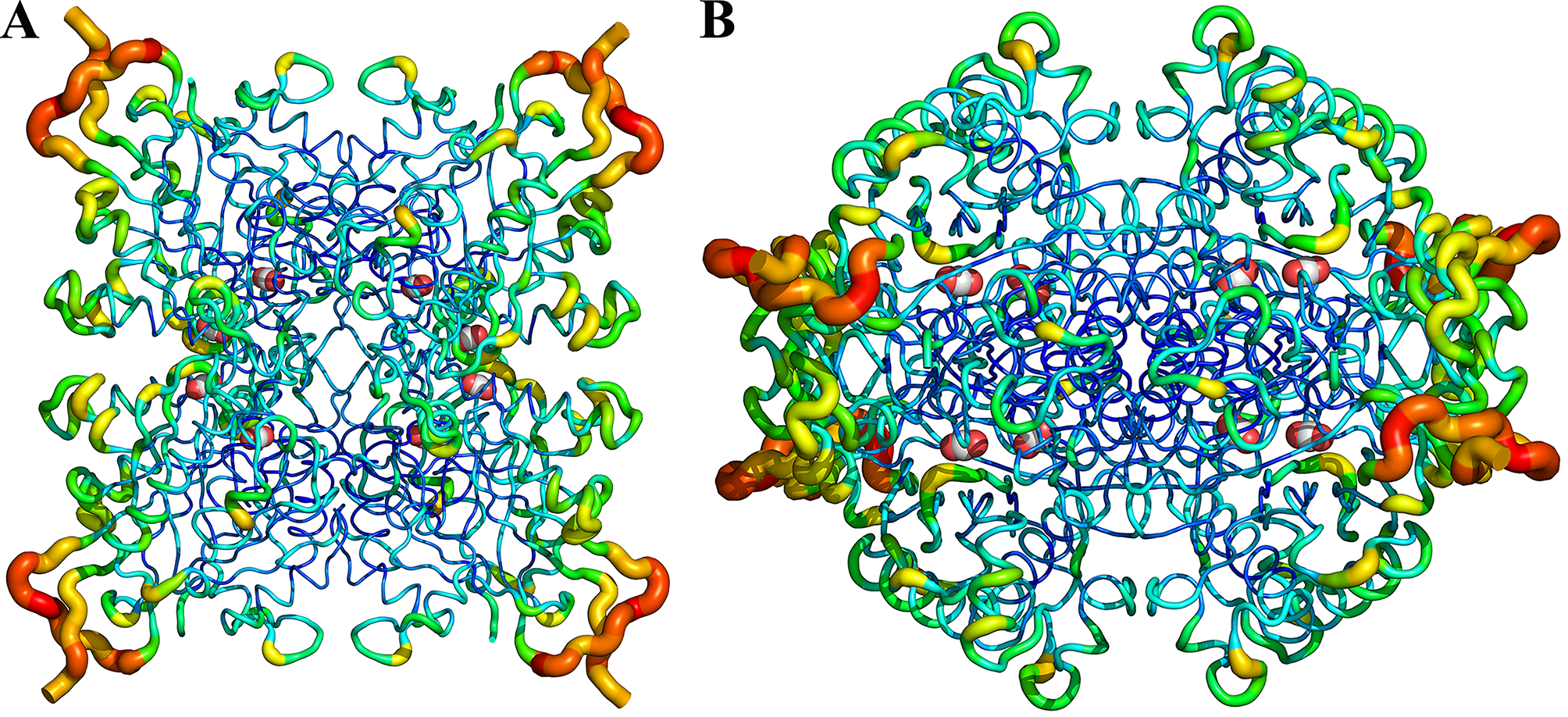


**Figure S4:** Locations of the ordered/disordered residues in TrzD tetramer. The main chains are color-ramped from blue to red for *B*-factors from approximately 25 to 93 Å^2^. The higher *B*-factor main chains are also shown in the thicker lines. Carbon dioxides are shown in spheres.


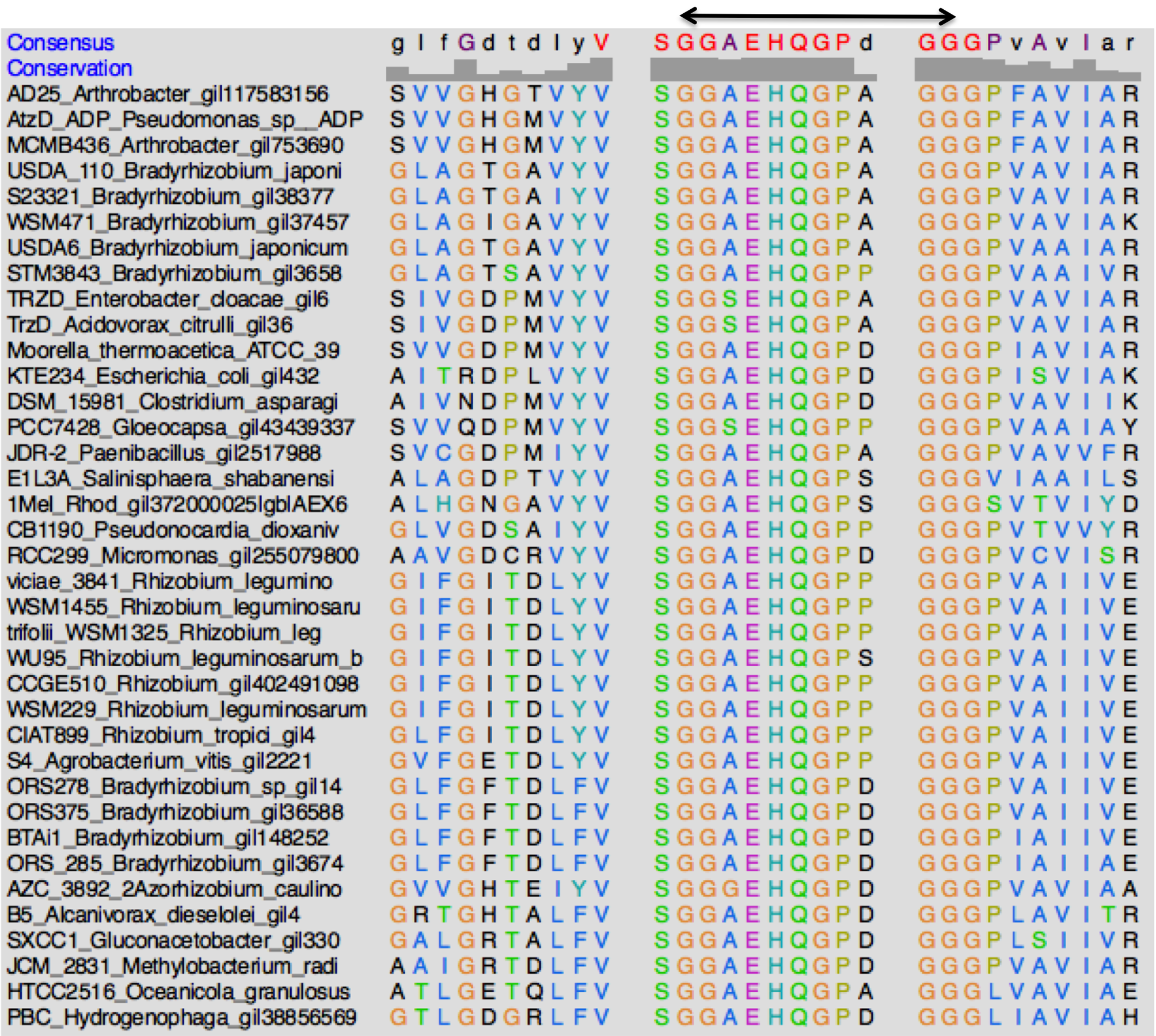


**Figure S5:** Domain 3 sequence alignment near metal binding flap. Conserve sequence of metal binding loop in cyanuric acid hydrolase/barbiturase family shows by an arrow.


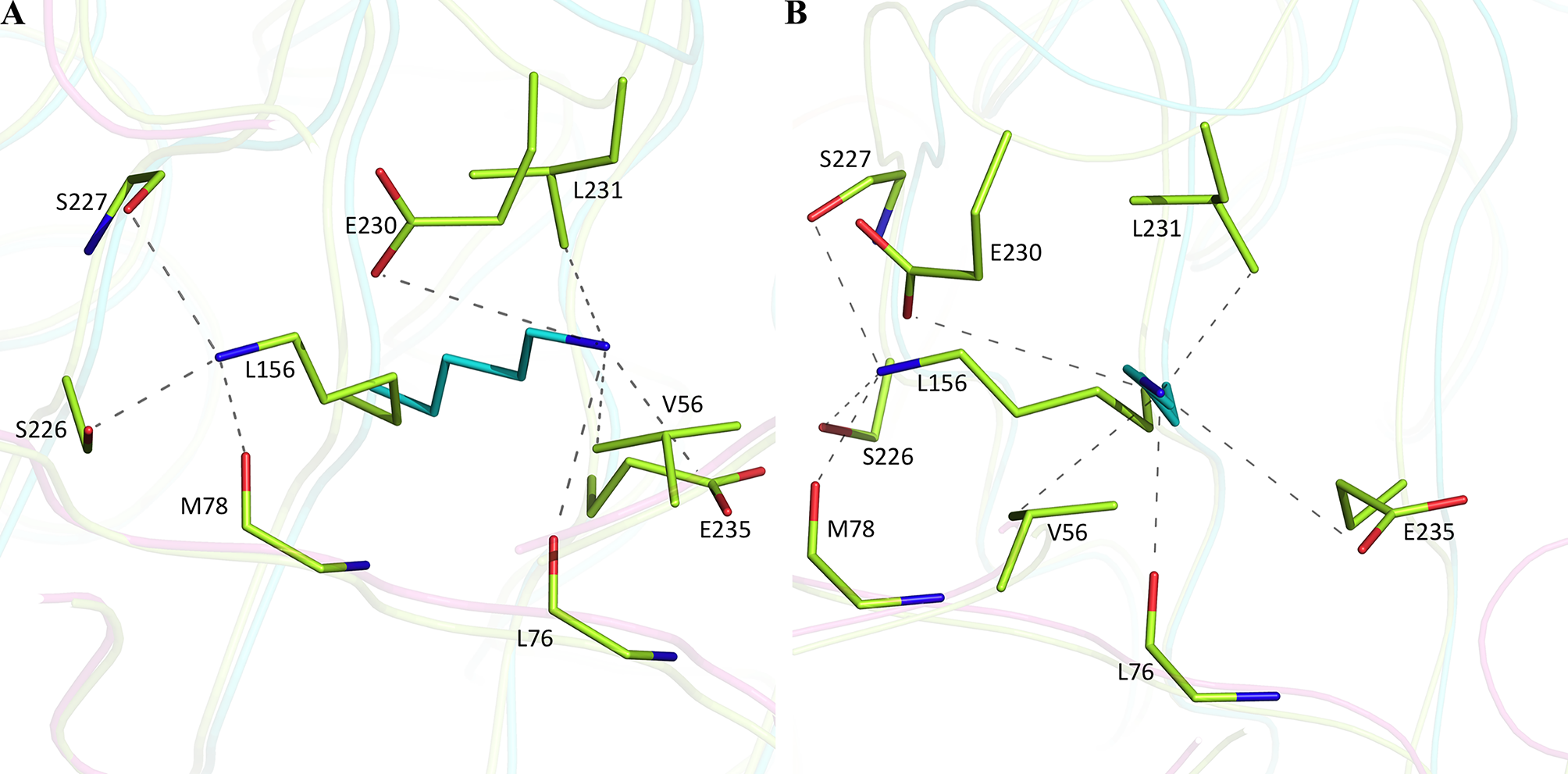


**Figure S6:** Superimposition of TrzD in cyan on ACAH in lime. **A)** L156 of ACAH (lime) H-bonded to two main chain carbonyl oxygens of S227 & M78 and with S226 OG. There is space for L156 to move in the orientation of TrzD L163 (shown in cyan), and if L156 moves nearest atom distances are 4 Å and more. **B)** 90° rotation along y-axis.

**
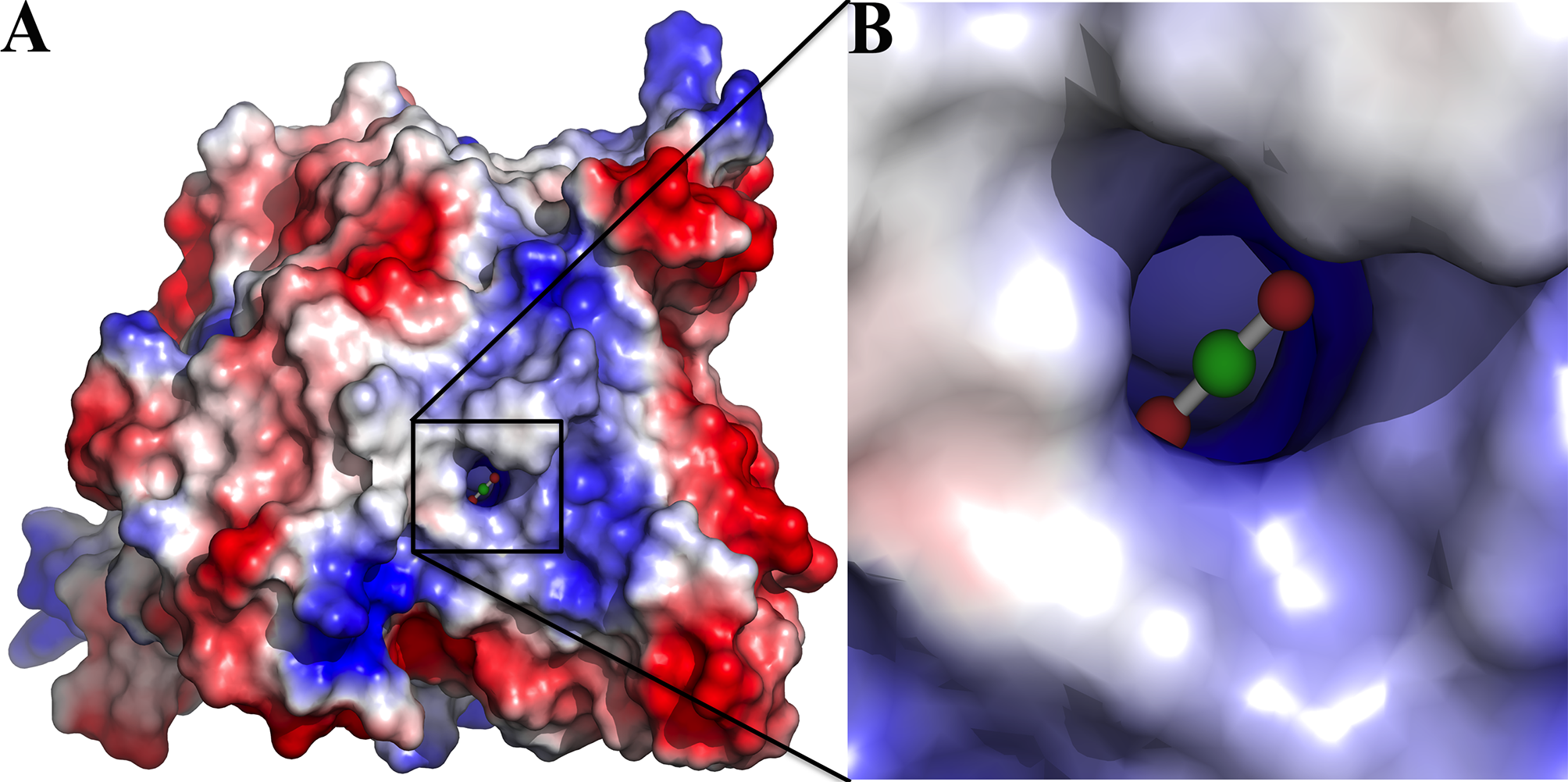
**

**Figure S7:** Electrostatic potential over TrzD surface. Carbon dioxide seen in the exit channel.
